# Tracheoinnominate Artery Fistula Treated With Endovascular Stent Graft at a Level I Trauma Center

**DOI:** 10.7759/cureus.9710

**Published:** 2020-08-13

**Authors:** Mujahed Laswi, Jorge Vega, Keith Jones, Lawrence Lottenberg

**Affiliations:** 1 General Surgery, Charles E. Schmidt College of Medicine, Florida Atlantic University, Boca Raton, USA; 2 Trauma and Acute Care Surgery, Florida Atlantic University/St. Mary’s Medical Center, West Palm Beach, USA; 3 Vascular Surgery, St. Mary's Medical Center, West Palm Beach, USA; 4 Surgery, Florida Atlantic University/St. Mary's Medical Center, West Palm Beach, USA

**Keywords:** tracheostomy, tracheoinnominate fistula, endovascular

## Abstract

Tracheoinnominate artery fistula could be a fatal complication of tracheostomy. Herein, we present the case of a 59-year-old male with sentinel bleeding around the tracheostomy with subsequent workup revealing a tracheoinnominate fistula. Subsequently, the patient was managed with an endovascular approach with a subsequent favorable outcome. We reported an alternative approach to the management of this catastrophic complication in patients who are at high risk for complications from conventional treatment approach.

## Introduction

Tracheoinnominate artery fistula (TIF) is one of the rare yet lethal complications of tracheostomy. Its incidence is reported at 0.1%-1% in different case series [1-4]. TIF most often presents as active bleeding at the tracheostomy site and/or from the tracheostomy tube. Standard management involves stabilization of the airway, hyperinflation of the cuff, and digital compression followed by emergent median sternotomy and ligation of the innominate artery [1,2,5]. Even with prompt management, there is a high mortality rate that reaches 100% in some series [1,2]. This high mortality is attributed to the difficulty of controlling the hemorrhage preoperatively, high infection rates postoperatively, and the patients often present with other comorbid conditions. Given the high mortality and morbidity rates with a standard operative approach, endovascular stenting has been described as an alternative intervention [4-9]. We present a case of a patient with TIF who was successfully treated with endovascular stenting of the innominate artery.

## Case presentation

A 59-year-old man with a history of cerebrovascular accident with right hemiplegia, dysphagia, and chronic ventilator dependence underwent open tracheostomy two years prior. The patient also had a history of median sternotomy for four-vessel coronary artery bypass grafting four years prior to this presentation. At the time of presentation, he had a no. 6, plastic, cuffless tracheostomy tube that has been in place for a year. The patient was transferred from an outside hospital due to a one-time episode of bright bleeding from the tracheostomy site and tracheostomy site. There was evidence of bleeding upon arrival, which spontaneously stopped. The patient was hemodynamically stable with a normal heart rate and blood pressure.

**Figure 1:**
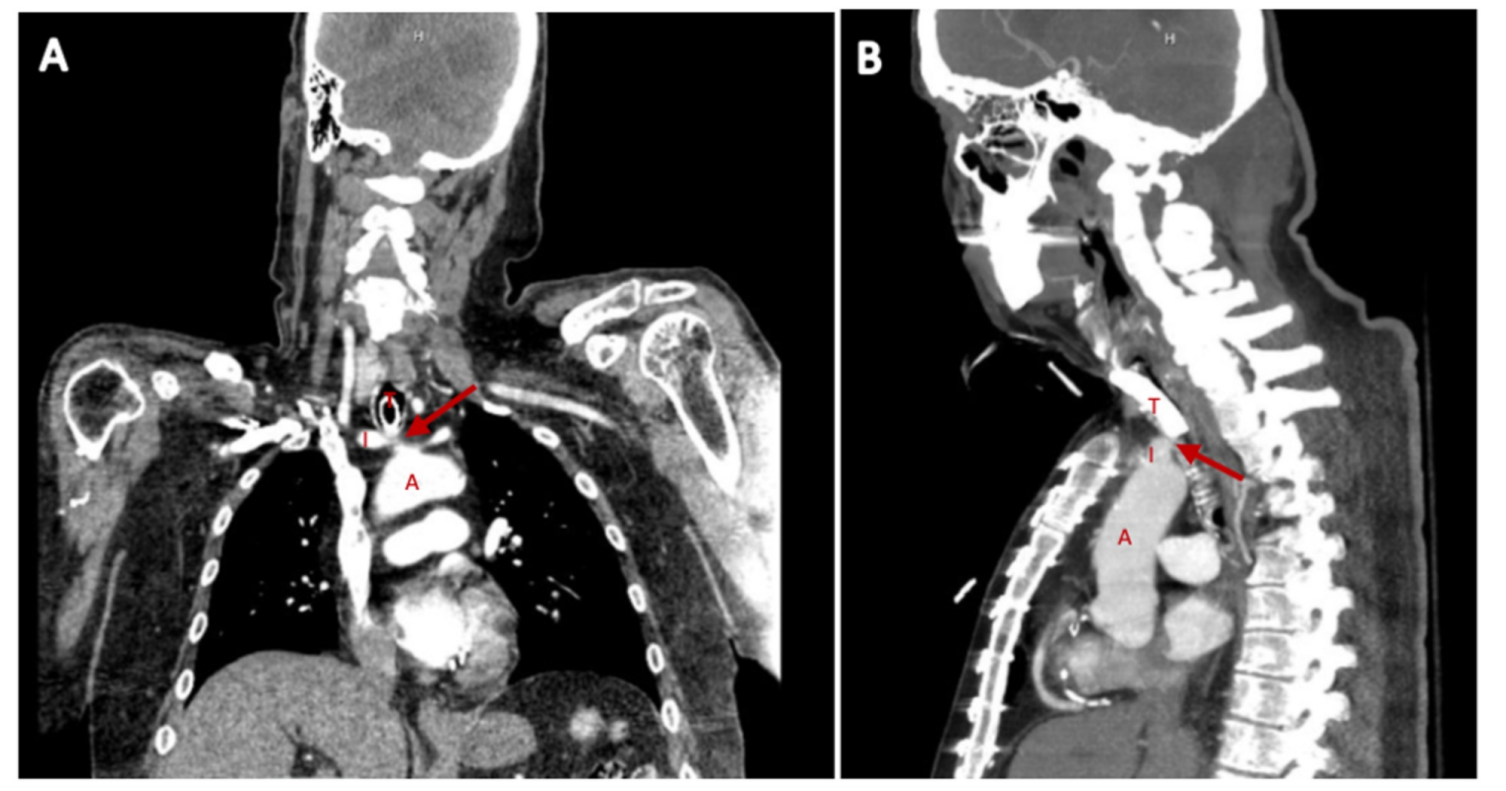
Coronal (A) and sagittal (B) views of CT angiogram of the chest and neck revealing a tracheoinnominate artery fistula (arrows). I: innominate artery, T: trachea with tracheostomy tube, A: aortic arch.

The patient underwent CT angiography of the neck and chest (Figure 1) revealing the innominate artery immediately anterior to the trachea and the tracheostomy tube. Due to a high index of suspicion for TIF, the patient underwent orotracheal intubation and bronchoscopy. Bronchoscopy revealed hyperemic mucosa of the anterior trachea around the tracheostomy site extending distally to the level of the carina, with blood clots at the carina, as well as in the right and left mainstem bronchi. There was evidence of paratracheal hematoma and blood at the tracheostomy site; however, there was no evidence of active bleeding.

**Figure 2:**
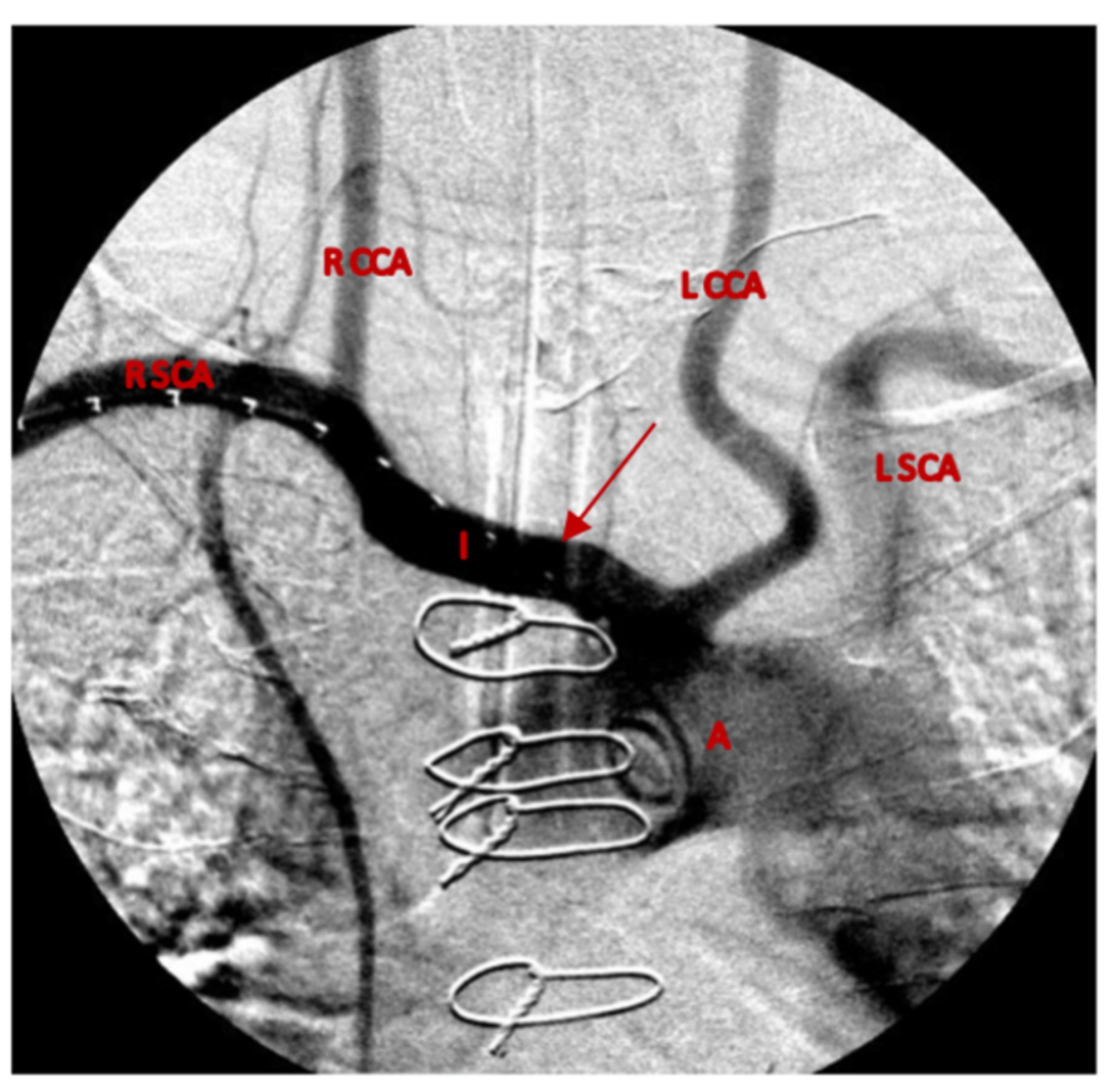
Diagnostic angiogram of the aortic arch and innominate artery revealing the artery immediately anterior to the trachea without a blush (arrow). This is also revealing the patient has a bovine arch variant with a common trunk of origin for the innominate artery and left common carotid artery. A: aortic arch, I: innominate artery, L CCA: left common carotid artery, R CCA: right common carotid artery, L SCA: L subclavian artery, R SCA: R subclavian artery.

With a history of median sternotomy for coronary artery disease and prior cerebrovascular accident, the patient was deemed at increased risk for open surgical treatment of his presumed TIF and underwent endovascular stenting of the innominate artery via a right brachial artery approach and using a cut-down technique and a 5-French sheath. The initial angiogram revealed the innominate artery crossing immediately anterior to the trachea without active hemorrhage (Figure 2). 

**Figure 3:**
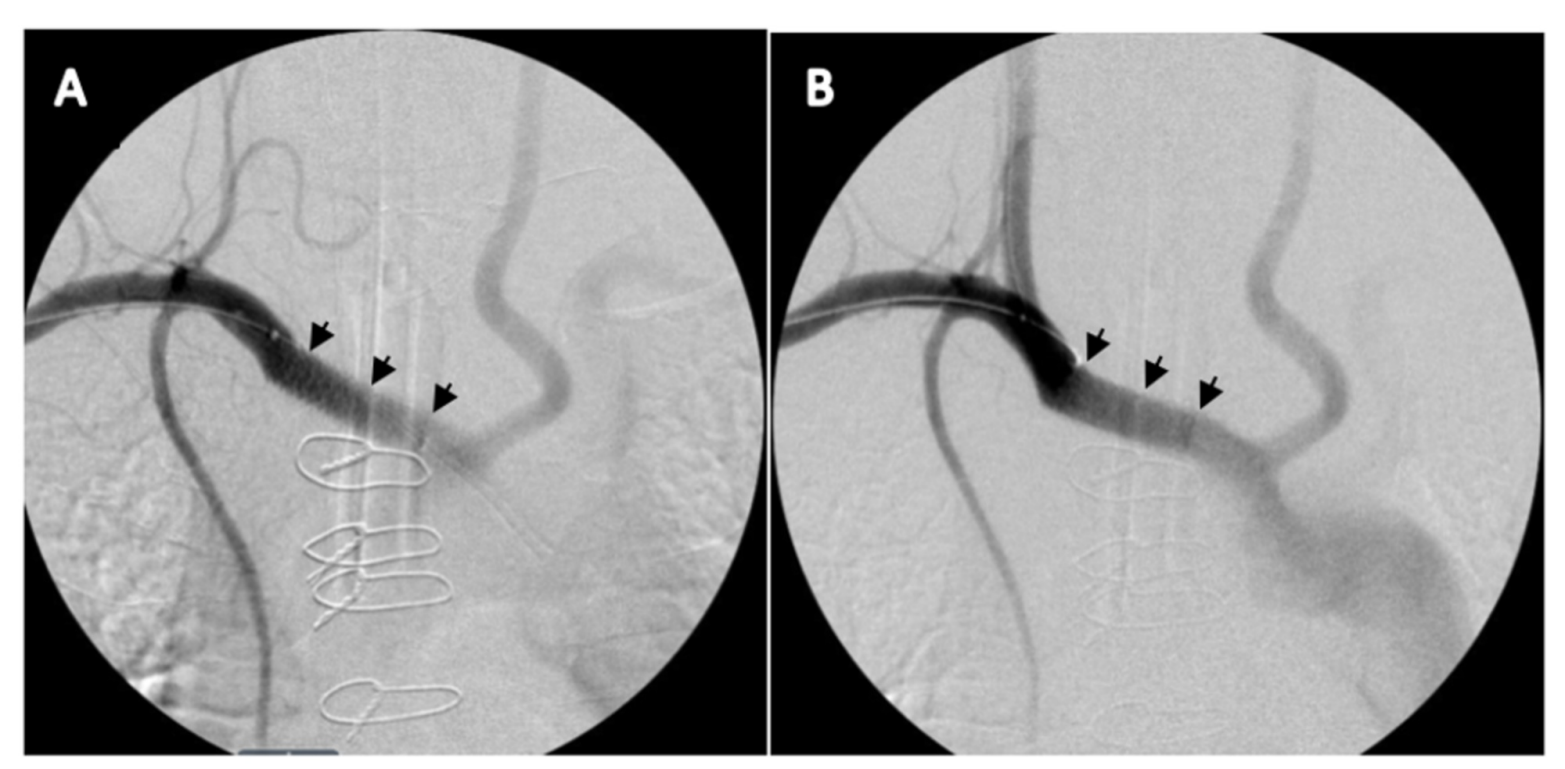
Angiography post stent placement with initial occlusion of the right common carotid artery (A). Balloon angioplasty at the origin of the right carotid artery displaced the stent and restored flow (B). Innominate artery completely excluded from trachea.

A 10 × 38 mm iCAST™ (Atrium Medical Corporation, Hudson, NH) covered stent was placed in the innominate artery (Figure 3A); this was somewhat challenging due to the presence of a bovine arch variant and concerns of covering the common origin of the innominate and left common carotid arteries. Post procedure angiography revealed sluggish flow through the right carotid artery. This was attributed to the stent covering part of the origin of the right carotid artery due to the stent’s lack of post-deployment predicted foreshortening (described in the Instruction for Use manual of the stent) [10]. To address this issue, balloon angioplasty of the origin of the right carotid artery was performed with a 5 × 20 mm balloon; this restored rapid flow to the right carotid artery (Figure 3B). The patient was monitored in the ICU for seven days without evidence of recurrent bleeding. The patient was extubated postoperatively on day 5. The patient was started on aspirin 81 mg postoperatively and was maintained on that dose.

## Discussion

TIF is a devastating complication of prolonged tracheostomy. The incidence has been reported at 0.1%-1% in different case series, and this may represent an underestimation due to underreporting and high early mortality rates [7,8]. New bleeding around an established tracheostomy site should prompt immediate evaluation for TIF. Approximately 35%-50% of patients develop a sentinel or intermittent bleed prior to life-threatening hemorrhage [6,7]. The leading cause for the development of TIF is thought to be pressure necrosis of the anterior tracheal wall caused by the tracheostomy tube and erosion into the innominate artery [6,8,11]. Factors that can increase the risk of fistula formation include positioning of tracheostomy below the fourth trachea ring, prolonged or repeated overinflation of the tracheostomy balloon, a high riding innominate artery, local infection, and previous radiation or operation [6,7].

Survival in patients with TIF requires immediate recognition/diagnosis followed by emergent hemorrhage control and expeditious occlusion of the fistulous tract. Emergent management involves securing the patient’s airway with endotracheal intubation and placement and maximal inflation of the ETT balloon below the level of the tracheostomy, and manual compression of the innominate artery at level of tracheal stoma/manubrium (Utley maneuver) [1,2]. This is followed by bronchoscopy and removal or endobronchial blood/clots and definitive therapy. Open operative management (median sternotomy and repair or ligation of the innominate artery) is fraught with high intra- and perioperative mortality rates related to technical difficulties in obtaining initial hemorrhage control and postoperative complications in this patient population [4].

Advancements in endovascular therapy may allow stenting to become a preferred initial management strategy. Safran et al. have described the use of covered endovascular stents in the treatment of isolated innominate artery pseudoaneurysms due to injuries from central venous access [12]. Endovascular stenting has been utilized in trauma patients with injuries to the innominate artery and great vessels [13,14]. Endovascular stenting has also been used for the treatment of TIF as a definitive treatment or as a bridge to definitive treatment [4,9,14]. Risks of endovascular management include stent infection, occlusion, and erosion into the trachea with rebleeding [4,9]. Most of the patients who developed these complications had evidence of previous sternotomy, chest wall deformity, or multiple comorbidities, also making them high-risk candidates for the surgical treatment [4-9].

Taechariyakul et al. have identified a total of 261 cases of TIF in a cohort analysis [7]. Of the 12.6% of cases that were treated endovascularly (covered stents or embolization of the innominate artery), survival was comparable between endovascular and surgical treatment, with a reported hazard ratio of 0.78 (CI 0.34-1.79).

In our case, the patient was deemed a high-risk candidate and underwent endovascular stenting with a covered stent. Different levels of challenges were noted, including bovine arch variant, occlusion of the right common carotid artery, and management of this complication endovascularly, which is first to be reported in the literature. The patient had a favorable outcome with resolution of bleeding. The patient will be monitored closely to assess long-term outcomes from endovascular stenting.

## Conclusions

Advances in endovascular interventions can be utilized in treating TIFs, a devastating complication of prolonged tracheostomy, with prohibitive operative mortality. Endovascular stenting might be the preferred approach in patients who are poor surgical candidates for surgical intervention. It is likely that it will become the preferred procedure for all patients with this complication. Long-term follow-up is required in these patients due to risks of stent placement, including infection, occlusion, and erosion. Several studies will need to be performed to assess morbidity and mortality in patients undergoing endovascular treatment of TIFs.
